# A case of malignant hyperthermia that was difficult to be differentiated from oral antipsychotic polypharmacy-associated neuroleptic malignant syndrome

**DOI:** 10.1186/s40981-016-0035-8

**Published:** 2016-06-02

**Authors:** Yoshiki Hara, Yutaka Hosoya, Ryo Deguchi, Shigehito Sawamura

**Affiliations:** Department of Anesthesiology, Teikyo University School of Medicine, 2-11-1 Kaga, Itabashi-ku, Tokyo, 173-8605 Japan

**Keywords:** Malignant hyperthermia, Neuroleptic malignant syndrome, Antipsychotic drugs

## Abstract

Malignant hyperthermia (MH) occurred during anesthesia with volatile inhalation anesthetics in a patient under treatment with multiple oral antipsychotic drugs and with a history of multi-acting receptor-targeted antipsychotic drug (MARTA)-induced elevation of serum creatine kinase (CK). Since the patient was considered to be at high risk for neuroleptic malignant syndrome (NMS) based on this history, differential diagnosis between MH and NMS was difficult at the time of onset. Later, the patient was found to be predisposed to MH based on abnormal high rate of the Ca^2+^-induced Ca^2+^ release (CICR). We concluded that MH was induced by the volatile inhalation anesthetics.

## Background

Patients under treatment with multiple antipsychotic drugs are at risk for multi-acting receptor-targeted antipsychotic drug (MARTA)-induced elevation of serum creatine kinase (CK), which is a risk factor for neuroleptic malignant syndrome (NMS). However, the symptoms of NMS are similar to those of malignant hyperthermia (MH). Here, we report a case of MH which was difficult to be differentiated from NMS during surgery for trauma.

## Case presentation

The patient was a 24-year-old man (76 kg/172 cm) with high-energy trauma caused by a fall from the 3rd floor of an apartment building. His injuries were second lumbar vertebral bursting fracture, left sacral and pubic fractures, right tibial and fibular fractures, and left calcaneal fracture. Multiple surgeries were planned, including posterior lumbar fusion and external fixation of the right lower limb in the first surgery, and subsequent open surgery for left calcaneal, right fibular, and right plafond fractures.

The patient had been diagnosed with developmental disorder at 9 years old, and was concomitantly diagnosed with schizophrenia at the time of injury. Risperidone (serotonin · dopamine antagonist (SDA)), Paliperidone (SDA), Aripiprazole (dopamine receptor partial agonist (DPA)), and Lorazepam (benzodiazepine anti-anxiety agent) were prescribed before injury, but the medication status immediately before injury was unclear. At 15 years old, oral Olanzapine (MARTA) elevated the CK level (33,040 IU/L) and the drug was withdrawn. There was no particular familial MH history.

The CK level was high (1,168 IU/L) on the first examination after injury, but this was considered to be due to trauma. Posterior lumbar fusion and external fixation of the right lower limb were performed 36 h after injury (operative time: 290 min). Anesthesia was induced with Propofol and maintained with Sevoflurane (90 min), followed by a switch to Desflurane (240 min) during surgery. Fentanyl and Remifentanil were administered for analgesia and Rocuronium was used for muscle relaxation.

Slow elevation of the end-tidal carbon dioxide concentration (ETCO_2_) occurred at 270 min after initiation of anesthesia (Sevoflurane exposure 90 min + Desflurane exposure 180 min) when wound closure was started. This was dealt with by increasing the minute ventilation volume from 6 L/min, but the rate of ETCO_2_ elevation rapidly increased 60 min later when the minute ventilation volume reached 10 L/min, and then became uncontrollable. Operation came to end just before this event, and Desflurane inhalation was terminated. One hundred twenty to 140 bpm tachycardia and more than 150/50 mmHg high blood pressure became marked in the same period, and the bladder temperature exceeded 38 °C and started to rise rapidly. Urine was slightly reddish and oliguria was evident. No muscle rigidity was observed.

Bladder temperature rose by 0.5 °C within 15 min. When this temperature reached 39 °C, the condition was clinically diagnosed as MH or NMS based on the rapid ETCO_2_ elevation, respiratory acidosis (pH 7.051, PCO_2_ 114.3 mmHg, HCO_3_^−^ 31mmoL/L, PO_2_ 391.9 mmHg), 190 bpm tachycardia, and 180/50 mmHg high blood pressure. Dantrolene was chosen for therapy. At the same time, cooling of the whole body was initiated and 10 mg Midazolam was injected intravenously for appropriate sedation. Preparation for Dantrolene administration required 15 min, and the ETCO_2_ and bladder temperature at initiation of Dantrolene were 111 mmHg and 40.2 °C, respectively.

**Fig. 1:**
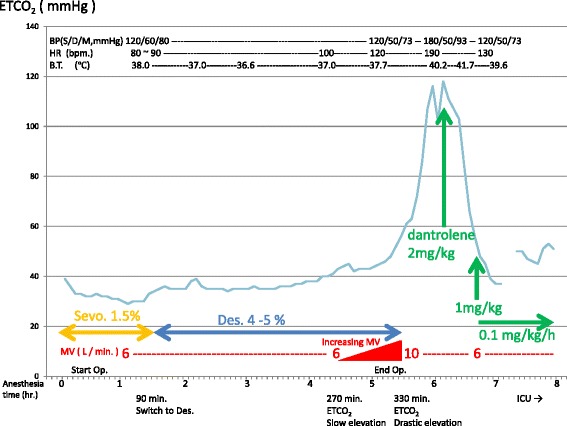
The anesthesia record of ETCO_2_, blood pressure, heart rate and bladder temperature. Slow elevation of ETCO_2_ was observed from 270 min. to 330 min. of anesthesia time, despite of increasing MV up to 10 L / min. 2 mg /kg I.V. dantrolene had a complete response for 111 mmHg uncontrollable ETCO_2_, turning the ETCO_2_ down to 60 mmHg after 25 min. One hundred ninety bpm tachycardia, and 180/50 mmHg blood pressure were observed with 41.7 °C peak bladder temperature

After initiation of 2 mg/kg Dantrolene, ETCO_2_ immediately started to decrease under the same ventilation conditions, and decreased to below 60 mmHg after 25 min. Bladder temperature rose even after Dantrolene administration reaching 41.7 °C, but the bladder temperature started to decrease at 25 min at 30 min after the first administration, 1 mg/kg Dantrolene was additionally administered, followed by continuous infusion at 0.1 mg/kg/h. Treatment in the operating room was completed with an ETCO_2_ of 37 mmHg, improved acidosis (pH 7.429, PCO_2_ 36 mmHg, HCO_3_^−^ 23.3mmoL/L, PO_2_ 525 mmHg), 130 bpm heart rate, 120/50 mmHg blood pressure and 39.6 °C bladder temperature at 50 min after treatment initiation. The anesthesia record of ETCO_2_, blood pressure, heart rate and bladder temperature were shown in Fig. 1 with the minute ventilation volume setting and Dantrorolene therapeutic timing chart. Treatment was continued in the intensive care unit and continuous Dantrolene infusion was completed after 270 min.

Vital signs were stable on postoperative day (POD) 1, but CK and serum K^+^ levels were high. Promotion of diuresis by volume loading was planned as a therapeutic strategy. Given the risks of heart failure and oxygenation failure, mechanical ventiration management under sedation with Propofol was continued till POD2 to ensure safety and then the endotracheal tube was removed. The CK level peaked at 4,916 IU/L on POD 2 and normalized to 148 IU/L on POD 16, the planned second-stage elective surgery was performed under total intravenous anesthesia (TIVA) with a combination of Propofol, Fentanyl, Remifentanil, and Rocuronium. The prescriptions for SDA and DPA were changed to Haloperidol and Flunitrazepam before surgery. No particular event occurred during this surgery.

**Fig. 2:**
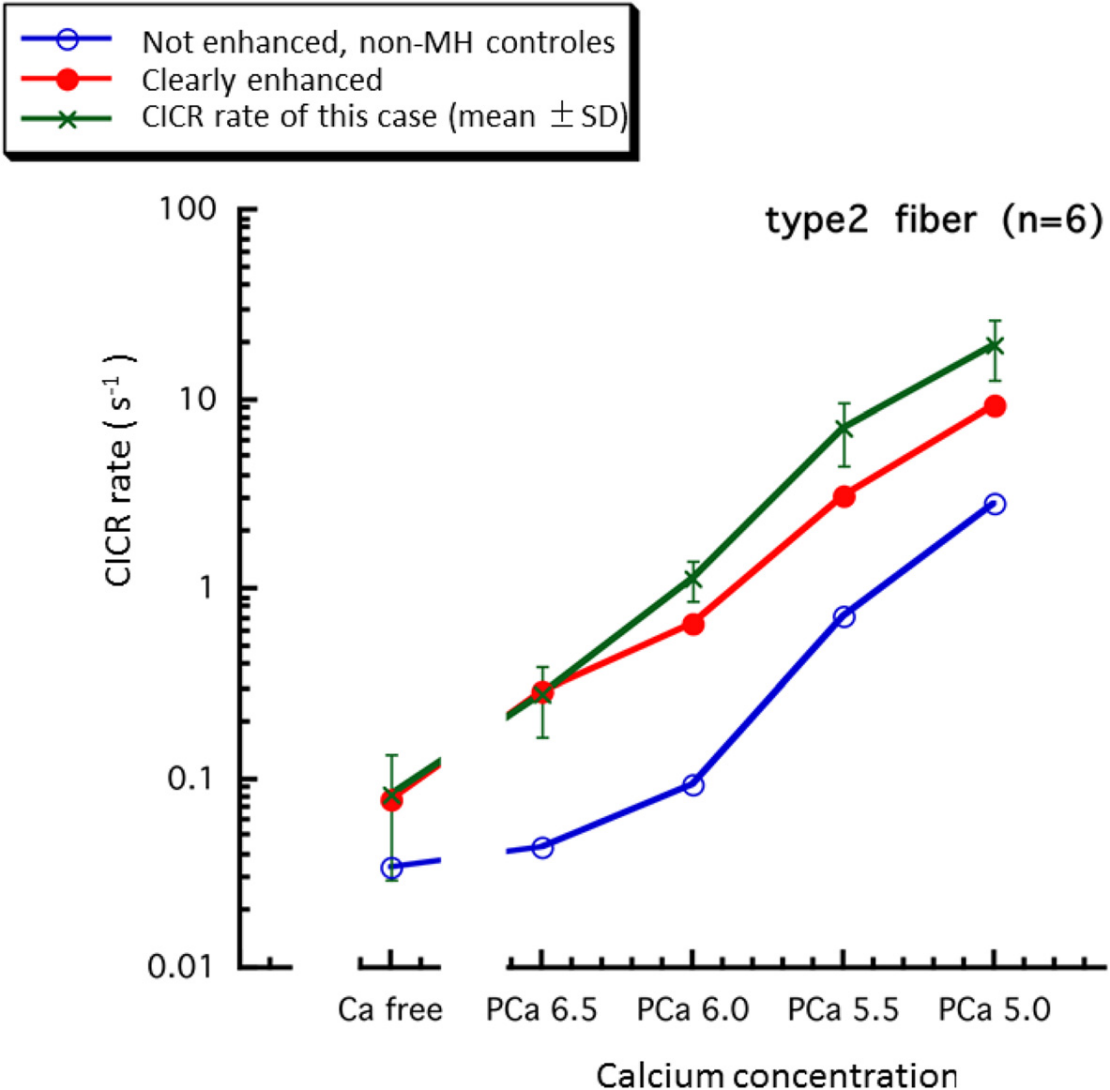
Comparison between CICR rate of this case and the reference values for malignant hyperthermia susceptibility diagnosis in Japan [14]. The data of this case are compatible with clearly high rate of CICR. Courtesy of Drs. *Hirosato* Kikuchi and Yasuko Ichihara (Saitama Medical University) for measuring CICR rate of this case

Eighteen months later, lumbar nails were surgically extracted under general anesthesia (TIVA) and biceps brachii muscle biopsy was simultaneously performed. The CICR rate was abnormally high (positive) using the skinned fiber method (Fig. 2), based on which the patient was definitively diagnosed with a predisposition to MH.

## Discussion

The patient had been treated with multiple antipsychotic drugs including SDAs and DPAs, which may cause NMS, and had a history of an oral MARTA-induced increase in CK, resulting in withdrawal of the drug. The medication status immediately before injury was unclear and the CK level was slightly high (1,168 IU/L). We considered these issues to be risk factors for NMS in preoperative evaluation [1]. On the other hand, the patient had no particular familial MH history and preoperative causal relationship between high CK level and MH was unclear [2]. Thus, we did not suspect a predisposition to MH.

Maintenance of anesthesia was initiated with Sevoflurane, but was switched to Desflurane during surgery with the aim of rapid arousal. Since MH developed after the switch to Desflurane, it appeared to be induced by Desflurane based on the time-course, but the possibility of induction by Sevoflurane cannot be excluded because late-onset MH has been reported [3]. No drug that may trigger MH was used other than the volatile inhalation anesthetics.

Regarding intraoperative management, the minute ventilation volume was increased gradually next 60 min after the detection of slow rise of ETCO_2_. During this period, body temperature was between 37 and 38 °C and we did not regard this as a sign of MH in spite of an ETCO_2_ rise prior to body temperature rises in many MH cases [4]. Since the criterion of body temperature elevation (≥0.5 °C increase within 15 min) was met when the body temperature rose to 39 °C with 190 bpm tachycardia and 180/50 mmHg high blood pressure, the drastic ETCO_2_ elevation was then clinically diagnosed as MH and Dantrolene was administered. Fortunately, the first-choice drug is Dantrolene for both MH and NMS. Thus, we administered this drug without hesitation, despite being unable to make a definitive diagnosis. Dantrolene showed remarkable effect of decreasing ETCO_2_ and body temperature, stabilizing hemodynamic status. However this effect had no value for therapeutic differential diagnosis between MH and NMS.

We reviewed the first surgery in order to differentiate between MH and NMS before the second-stage elective surgery. The symptoms observed during the first surgery were clinically diagnosed as MH. In MH, the drastic and uncontrolled increase in oxidative metabolism in skeletal muscle and the elevation of carbon dioxide production are important symptoms clinically compatible with this case. However, occasionally carbon dioxide may be elevated due to increased production in conditions such as thyroid storm, hyperpyrexia, shivering, MH and NMS [5, 6]. Therefore MH is not diagnosed only by the drastic elevation of ETCO_2_. The definite diagnosis of MH requires muscular testing, in vivo contracture test or CICR test. On the other hand, no laboratory test is definitively diagnostic for NMS and the diagnosis depends on medical history and is finally made by exclusion of other diagnosis [7, 8], so oral antipsychotic polypharmacy-associated NMS could not be ruled out in this case without definite MH diagnosis made by muscular testing. Therefore, we consulted the psychiatry department during preoperative planning, and got their consensus that the NMS possibility couldn’t be denied. The prescriptions for SDA and DPA were changed to Haloperidol and Flunitrazepam before surgery, while keeping in mind that most antipsychotic drugs may cause NMS.

On the assumption that he was MH patient, in order to determine the appropriate timing for the second-stage elective surgery, we searched the literature to investigate the interval between surgeries in cases of MH, but there has been no report describing an interval that ensures safety. Due to the multiple traumas, it was considered desirable to perform the second-stage elective surgery as soon as possible to prevent long-term functional disorder, separately from life support. Since the treatment outcome for MH is evaluated based on improvement of clinical symptoms and recovery of destroyed muscle tissue, using the CK level as an index [9], we determined that surgery could be performed after the CK level decreased to ≤200 IU/L.

No problem occurred in the second-stage elective surgery performed under TIVA. Thus, we concluded that if MH had occurred the symptoms observed in the first surgery were induced by the volatile inhalation anesthetics, whereas if NMS had occurred, the causes were the oral SDAs and DPAs that were withdrawn before the second surgery. Examination of the predisposition of the patient to MH was necessary to make a definite diagnosis.

After completion of all treatments for traumas, we planned a muscle biopsy to make a definite diagnosis. Since 2–3 months are required for regeneration and recovery of muscle after onset of MH [10], an early procedure of muscle biopsy is generally performed under local anesthesia. However, an examination under local anesthesia was considered difficult for this patient based on his background. Thus, muscle biopsy was performed during surgical lumbar nail extraction under general anesthesia (TIVA) after 18 months from the injury. The CICR rate was abnormally high using the skinned fiber method and this allowed a definite diagnosis of a predisposition to MH.

The mechanisms of MH and NMS differ [11], but the clinical symptoms are similar. In a surgical case in which a patient with a risk factor for NMS develops MH-like symptoms during general anesthesia with a volatile inhalation anesthetic, it is difficult to immediately differentiate between MH and NMS. This was the situation in our case.

Regarding the definite diagnosis, the CICR rate measured using the skinned fiber method is commonly high in MH patients and their families, but it does not occur in NMS patients [12]. Therefore, muscle biopsy for the definite diagnosis of MH was very useful in our clinically suggestive MH patient. However, cross-reactivity between MH and NMS exists on in vivo contracture test and conflicting results have been reported regarding the prevalence of MH susceptibility among NMS patients [13], so in vivo contracture test should be interpreted cautiously for the differential diagnosis between MH and NMS.

The predisposition to MH and the induction of MH by volatile inhalation anesthesia, but not in the second surgery using TIVA, indicate that MH in our patient was induced by volatile inhalation anesthetics.

## Conclusions

We encountered a patient who developed MH that was difficult to be differentiated from NMS due to oral antipsychotic polypharmacy. Subsequently, the patient was definitely diagnosed with a predisposition to MH based on abnormal high rate of the CICR. Because the patient had no MH episode in the second-stage elective surgery performed under TIVA, we concluded that the MH was induced by volatile inhalation anesthetics.

## Consent

Written informed consent was obtained from the patient and the parents for publication of this case report and any accompanying images. A copy of the written consent is available for review by the Editor-in-Chief of this journal.
